# Iodine Clusters in the Atmosphere II: Cluster Formation Potential of Iodine Oxyacids and Iodine Oxides

**DOI:** 10.1021/acsomega.5c02147

**Published:** 2025-06-03

**Authors:** Morten Engsvang, Jonas Elm

**Affiliations:** Department of Chemistry, 1006 Aarhus University, Langelandsgade 140, 8000 Aarhus C, Denmark

## Abstract

Iodine-driven nucleation is thought to be a significant source of new particle formation, especially in marine and polar regions. Despite numerous studies, the mechanism is still not fully understood. To shed further light on this, we apply ZORA-DLPNO–CCSD­(T_0_)/TZVPP//ωB97X-D3BJ/aug-cc-pVTZ-PP to calculate the thermochemistry of iodine-containing clusters up to tetramers and simulate the cluster formation potential for several nucleation paths using the atmospheric cluster dynamics code (ACDC). We find that iodine oxyacid–amine nucleation can be competitive with sulfuric acid–amine nucleation if iodic acid is present in a 10:1 ratio compared to sulfuric acid. Therefore, the importance of the iodine-driven pathway is regionally dependent. Likewise, we find that increasing the relative humidity from 34 to 73% only changes the cluster formation potential by a factor of 2. Nucleation pathways consisting of only iodic and iodous acid are unable to explain the relative nucleation rates previously observed in experiments. In contrast, the simultaneous nucleation of iodine oxides, assisted by iodine oxyacids, is better able to describe the trend. This indicates that a nucleation pathway starting with iodine oxides is more likely to be able to explain observed particle numbers. However, this current model does not include all of the hydrates of the clusters and does not account for the hydrolysis reactions of the iodine oxides. This would need to be incorporated in future studies.

## Graphical abstract

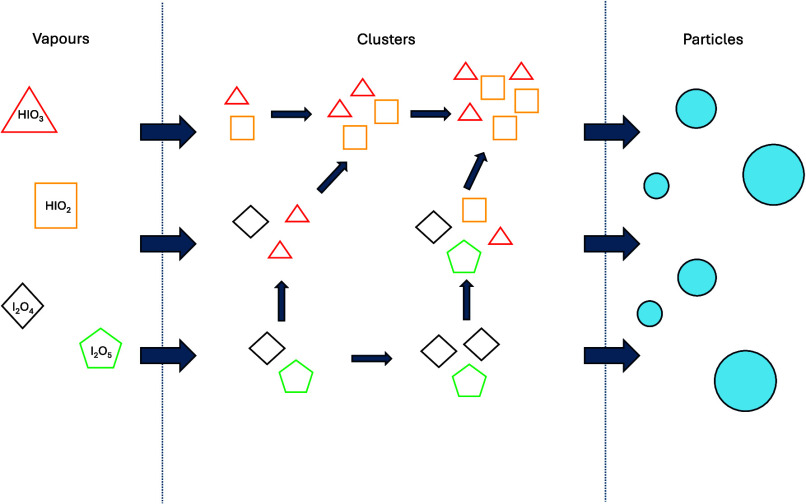

## 1 Introduction

The fraction of cloud condensation nuclei (CCN) formed via new particle formation (NPF) can vary significantly across the globe, but in many cases, it can exceed 50% of the total CCN concentration.^1^

The onset of NPF is the formation of small clusters from atmospheric vapors that grow further by the condensation of vapor or coagulation of clusters. However, the exact mechanism describing which vapors participate during the different processes is still poorly understood.^2^

It is now well-established that polar regions are warming significantly faster than other parts of the globe,^3^ with regions like the Arctic warming 4 times faster, with some local warming reaching 7 times faster warming compared to the global average.^4^ This warming is affected by the changing Arctic climate in a strong feedback loop. Formation of aerosols is a significant contributor to this feedback loop, both directly and indirectly through the scattering of light and acting as CCN. In the Arctic, particles ranging from 50–100 nm can act as CCN to form low-level clouds that can contribute to additional warming.^5,6^ Locally formed CCN is primarily relevant for the Arctic summer,^7^ where spring and fall is dominated by anthropogenic sources.^8^

Proper speciation of NPF is difficult to achieve with experimental techniques. This is due to the relatively weak intermolecular bonds holding the clusters together, which can easily be broken upon ionization.^9^ Determining the cluster composition beyond the elemental composition is also challenging and can be a hindrance to interpretation, where several distinct chemical isomers can have identical elemental composition. Thus, quantum chemical studies can be necessary to aid in the interpretation by calculating the cluster structure and thermodynamics.

Iodine-containing compounds are ubiquitous in the atmosphere, but are found in large concentrations in marine, coastal, and polar environments.^10–14^ Studies have shown that high concentrations of iodine-containing compounds leads to NPF in Arctic and marine environments.^11,13,15,16^ Previous computational studies have been carried out on iodine-driven cluster formation leading to NPF. The focus has primarily been on iodic acid (HIO_3_) and iodous acid (HIO_2_),^2^ both on their own,^16–19^ but also together with other common nucleation precursors such as sulfuric acid,^20^ methanesulfonic acid,^21–24^ ammonia,^25^ and dimethylamine.^26,27^ Especially, iodic acid assisted by various bases has been shown to be a potent source of new particles.

Experimental studies^16,28–33^ have shown that iodine on its own can nucleate. However, the exact contributions of different iodine-containing compounds are still not fully understood due to disagreements on the nucleation pathway and the mechanism of how and where iodine-containing nucleation precursors are formed. For example, there is only one significant proposed gas-phase formation mechanism of HIO_3_,^14^ and whether HIO_3_ is even formed in the gas-phase, or if it is formed at the ionization has been discussed.^34,35^ For the other relevant compounds, such as HIO_2_, and the oxides,^35^ the formation mechanisms still remain unexplained.

Nucleation studies are split between an iodine oxyacid path (iodic acid and iodous acid)^11,14–16,18,19,36^ and an iodine oxide pathway.^33,35,37,38^ Thus, a definitive conclusion is yet to be reached. The oxyacids and oxides are linked through hydrolysis reactions, and new experiments have shed some light on the effects of hydration on iodine-driven nucleation;^36^ however, a comprehensive mechanistic understanding is still missing.

In this paper, we study the competition between the iodine oxyacid and iodine oxide pathways for neutral iodine-driven nucleation using scalar relativistic quantum chemical methods. This is in contrast to many of the cited computational studies, which only take relativistic effects into account through the use of pseudopotentials. We study the cluster formation potential by simulating the cluster dynamics of cluster formation up to tetramers. This was done with the aim of shedding light on the compounds that are important for the initial steps of iodine-driven nucleation. Furthermore, we also study the strength of iodine-driven nucleation in relation to the already extensively studied sulfuric acid-driven nucleation.

## 2 Computational Details

### 2.1 Studied Systems

This study explores clusters of the type: (acid)_1–2_(base)_1–2_, where acid can be a combination of iodic acid (IA), iodous acid (IsA), sulfuric acid (SA), methanesulfuric acid (MSA), nitric acid (NA), or formic acid (FA), with the restriction of at least one iodine-containing monomer being present. The monomer and dimer structures are taken from our previous study.^39^ Base denotes any combination of water (W), ammonia (AM), methylamine (MA), dimethylamine (DMA), and trimethylamine (TMA). Furthermore, we also calculate the structures of the form (IA/IsA)_3–4_, such that these combined with the other clusters comprise a pure iodine oxyacid nucleation path. Here, (IA/IsA) denotes that it can be any combination of IA and IsA. Due to difficulties with representing the acid–base interaction between IA and IsA in our sampling, we have reoptimized and recalculated the structures from the study by Zhang et al.;^17^ however, for the (IA)_3–4_ structures, we found better structures, which we use instead.

Finally, we reoptimize and recalculate the SA–amine structures from the study by Elm^40^ which are on the form (SA)_0–2_(base)_0–2_, where base denotes AM, MA, DMA, and TMA. The (AM)_1_(MA)_1_ system optimized to an unstable cluster configuration and was therefore resampled according to the workflow described for the iodine-containing clusters.

We also include the dehydration products of the iodine oxyacid clusters; see the reactions in eqs 1 and 2, that is, iodine pentoxide (IP) and iodine tetroxide (IT).

1 $$I_{2}O_{5}+H_{2}O⇋2{\mathrm{HIO}}_{3}$$

2 $$I_{2}O_{4}+H_{2}O⇋{\mathrm{HIO}}_{3}+{\mathrm{HIO}}_{2}$$

For all clusters containing the (IA)_2_ or (IA)_1_(IsA)_1_ dimers, we include the equivalent clusters where these are replaced by (IP)_1_(W)_1_ and (IT)_1_(W)_1_ respectively as shown in the reactions in eqs 1 and 2. This results in the addition of clusters consisting of (IT/IP)_1–2_(W)_0–3_. However, because many of the reaction rates have yet to be determined, we do not include the dehydration/hydration reactions in our kinetics scheme. The hydrolysis reaction presents a reaction barrier in the gas-phase,^41^ but the reaction barrier or lack thereof in the cluster has not been determined. The studied clusters are split into three nucleation pathways:

- “pure oxyacid”: (IA/IsA)_0–4_
- “oxyacid-assisted oxide”: (IT/IP)_0–2_(W)_0–3_ and (IT/IP/IA/IsA)_1–2_(W)_0–2_
- “oxyacid + mixed acids”: (IA/IsA/SA/MSA/NA/FA)_0–2_(W/AM/MA/DMA/TMA)_0–2, with the restriction that the clusters need to contain at least one iodine-containing monomer: IA/IsA

Excluding the clusters reoptimized from Elm^40^ and Zhang et al.,^17^ and the dimers carried over from our previous study,^39^ we have calculated 299 new cluster structures in this study. These are available on the ACDB,^42^ and a direct link is given in the SI overview. Some of the calculated clusters did not end up being used in the ACDC analysis but are still supplied in the ACDB.

### 2.2 Workflow

The configurational sampling workflow follows closely the one used in our previous study on iodine in the atmosphere, but with slight modifications.^39^ We use the JKCS2.1 ^43^ program to manage the calculations. We follow the overall workflow set out by Kubečka et al.,^44^ where, for each type of cluster, an initial ensemble of structures is generated using ABCluster^45,46^ using the CHARMM force field.^47^ This was done using an initial population of 3000 structures, running for 200 generations using 4 “scout bees,” with 1000 structures saved for further calculations. We employed all combinations of ionization states for the monomers in the calculations. These structures were subsequently optimized using GFN1-xTB^48^ in the xTB program version 6.4 ^49^ as a preoptimization step. A uniqueness test was used on the GFN1-xTB structures to reduce the number of redundant calculations, thereby reducing the computational load. These are chosen, such that they are as far from each other in the chemical space by comparing the gyration radius, electronic energy, and dipole moment. All further calculations were carried out in ORCA 5.0.4,^50^ and for the final results, we optimized the structures at the ωB97X-D3BJ level of theory with an aug-cc-pVTZ basis set for all atoms except iodine. Iodine atoms were treated using the SK-MCDHF-RSC^51^ pseudopotential for the inner 28 electrons, with the outer electrons treated with the aug-cc-pVTZ-PP basis set. This treatment is simply denoted as aug-cc-pVTZ-PP from hereon.

For the final results, we also calculated single-point energy corrections on the five lowest free energy structures at the ωB97X-D3BJ/aug-cc-pVTZ-PP level with ZORA-DLPNO–CCSD­(T_0_). ZORA-DLPNO–CCSD­(T_0_) means a DLPNO–CCSD­(T_0_)^52,53^ calculation using the ZORA^54^ scalar relativistic Hamiltonian, where implicitly, tight PNO settings^55^ were applied for all relevant calculations. This was carried out using the ma-ZORA-def2-TZVPP basis set for all noniodine atoms and with the SARC-ZORA-TZVPP for iodine, which will be denoted as TZVPP. The quasi-harmonic approximation^56^ was used in the treatment of vibrational frequencies below 100 cm^–1^.

Spin–orbit coupling was not applied to the clusters, but, based on our previous study,^39^ the effect on IA and IsA is a small overestimation of the stability of the clusters on the order of 0.1 kcal mol^–1^, while the effect on IP and IT is a larger underestimation of the stability on the order of 1 kcal mol^–1^.

The result of any quantum chemical methodology will be sensitive to the size of the basis set applied, especially for atoms with a large number of electrons such as iodine. In our previous study,^39^ specifically reported in Table S8 of the Supporting Information, we studied the basis set convergence for small iodine-containing clusters, containing both iodine oxyacids and oxides. Using RI-MP2, we studied the effect of increasing the basis set size from double ζ, to triple ζ, quadruple ζ, and pentuple ζ. We found that the mean change from triple to quadruple was a stabilization of −0.18 kcal mol^–1^, and from quadruple to pentuple was a further stabilization of −0.2 kcal mol^–1^. However, this was dependent on the exact iodine compound present, and the iodine oxyacids were well converged at triple ζ, with the iodine oxides “driving” this mean change toward increased stability. From this, we conclude that the stability of the iodine oxyacids is well described at this level of theory, while the stability of the iodine oxides is slightly underestimated. This is in addition to the effect of underestimating the stability stemming from the spin–orbit coupling.

Binding free energy and thermal contributions to the binding free energy are defined as in eqs 3 and 4, where *i* denotes the components of a given cluster. In the first equation, we define the binding free energy as the total free energy of the cluster with the sum of the monomer free energies subtracted. In the second equation, we define the binding free energy as a sum of an electronic binding energy contribution and a thermal binding energy correction. In our scheme, the thermal binding energy correction includes both the changes in thermal effects and changes in vibrational zero-point energy.

3 $$\Delta G_{\mathrm{bind}}=G_{\mathrm{cluster}}-\sum_{i}G_{i}$$

4 $$\Delta G_{\mathrm{bind}}=\Delta E_{\mathrm{bind}}+\Delta G_{\mathrm{bind}}^{\mathrm{thermal}}$$

### 2.3 Cluster Formation Dynamics

The cluster formation dynamics are calculated using the atmospheric cluster dynamics code (ACDC).^57,58^ Size-dependent coagulation losses were used in the simulations (cs_exp = −1.6, cs_ref = 10^–3^).^59^ For simulations involving iodine, a collision enhancement factor of 2.4 was applied to all cluster collisions to take into account the dipole–dipole interaction based on the calculations by Zhang et al.^17^ A collision enhancement factor of such magnitude has previously been suggested for other systems.^60–62^ Thus, it was applied to all simulations except for the SA–amine simulations. It should also be noted that ideally an enhancement factor should also be used for the SA-amine systems; therefore, we underestimate the favorability of SA-driven nucleation compared to iodine-driven nucleation.

We report the potential formation rate *J*_potential_ of outgrowing clusters, which is the sum of the fluxes to larger clusters that are considered stable enough to grow out of the simulation scheme. That is, *J*_potential_ corresponds to the potential to form larger clusters. This is important to note as this means that *J*_potential_ should not be used directly as a measure of the NPF rate. Studying cluster dynamics with only up to 4 molecules can lead to artificially higher values as the critical cluster size/area may not be properly covered by the cluster dynamics simulations. Kubečka et al.^59^ reported that using a simulation scheme of (acid)_0–2_(base)_0–2_ instead of (acid)_0–4_(base)_0–4_ for SA–multibase clusters at low temperatures and the concentration ranges used in this work yields *J*_potential_ that are between 1 and 2 orders of magnitude higher while the trends in formation rates remain intact. However, the rates obtained after this correction will still be overestimated compared to measured nucleation rates; for example, the largest 4 × 4 cluster reported by Zhang et al.^17^ is (HIO3)_4_(HIO2)_4_, which approaches a diameter of 1.5 nm, while measurements typically start at 1.7 nm. Thus, an overestimation of the nucleation rate is still to be expected.

Furthermore, for the oxyacid-assisted oxide nucleation, the collision of (IP)_1_(IsA)_1_ and similar oxide + oxyacid clusters was allowed to grow out of the system, even if they only collided with another oxyacid. The resulting cluster is thus effectively only a tetramer and will lead to an overestimation of the nucleation potential. However, this is unlikely due to the high stability of these clusters at this level of theory combined with the observation that the stability of iodine oxide clusters is still underestimated compared to iodine oxyacids, even at this level of theory, based on the observed basis set convergence as discussed in Section 2.2.

We define the threshold for a significant nucleation to be at a nucleation rate of 1 cm^–3^ s^–1^. This threshold is based on a qualitative assessment of atmospheric observations of nucleation events, see, for example, Figure 1 of Almeida et al.,^63^ where most observations of nucleation do not go below 1 cm^–3^ s^–1^. Therefore, with the aforementioned limitations, we would need to have a formation potential of at least an order of magnitude higher than this to be sure that the real nucleation rate is significant. This means that for our cluster sizes, a significance threshold of 10–100 cm^–3^ s^–1^ is applied to our calculations of the potential nucleation rate; with 10 cm^–3^ s^–1^ being a minimum threshold and 100 cm^–3^ s^–1^ for it to be certain.

The outgrowing clusters were chosen as all of the 5 molecule clusters that can be constructed from collision by a single of the monomer molecules included in the simulation. However, due to the size of IT and IP, these are effectively treated as 2 molecules, i.e., we allow smaller clusters like the IT-IT, IT-IP, and IP-IP to also be outgrowing clusters. This is because both IT and IP can be converted to two acid molecules by a simple reaction with water.

The concentrations used in the simulations are given in Table 1 unless otherwise specified. If water is included, relative humidity was set to a lower limit of 34% and a higher limit of 73% based on the relative humidities used by He et al.^16^ The concentrations of IsA, IT, and IP were set as a fraction of the IA concentration. IT and IP was set to 1% of IA based on the relative concentration of IT reported by He et al.,^16^ while the IP concentration was assumed to be identical to IT due to a lack of measurements. The relative concentration of IsA varied with temperature, with a relative concentration of 1% of IA at 283 K, and 3% of IA at 263 K based on the data reported by Zhang et al.^17^ The ranges for SA and IA is based on reported concentrations in the Arctic^15^ and in marine areas.^11^ FA, MSA, and NA concentrations were chosen to be consistent with previous studies.^64,65^

**Table 1:** Low and High Estimates of Gas Precursor Concentrations^a^

chemical species	low concentration	high concentration
	bases	
AM	10 ppt	10 ppb
MA	1 ppt	10 ppt
DMA	1 ppt	10 ppt
TMA	1 ppt	10 ppt
	acids	
FA	2.46 × 10^11^ cm^–3^	2.46 × 10^11^ cm^–3^
MSA	10^6^ cm^–3^	10^6^ cm^–3^
NA	2.46 × 10^11^ cm^–3^	2.46 × 10^11^ cm^–3^
SA	10^5^ cm^–3^	10^7^ cm^–3^
IA	10^6^ cm^–3^	10^8^ cm^–3^
IsA	10^4^ cm^–3^	3 × 10^6^ cm^–3^

a The following abbreviations are used: ammonia (AM), methylamine (MA), dimethylamine (DMA), trimethylamine (TMA), formic acid (FA), methanesulfonic acid (MSA), and nitric acid (NA).

## 3 Results and Discussion

### 3.1 Cluster Structures

A selection of cluster structures can be found in Figure 1, and these structures are representative of some of the main outgrowing clusters identified later. It can be observed that the pure IsA cluster favors I–O halogen bonds above forming any hydrogen bonds, where all of the hydrogens are pointed outward from the structure. The interaction between IA and IsA introduces hydrogen bonding and leads to an acid–base interaction between the acids, as previously observed by Zhang et al.^17^ However, it can be seen that IsA still prefers intermolecular halogen bonds between IsA molecules.

**Figure 1:**
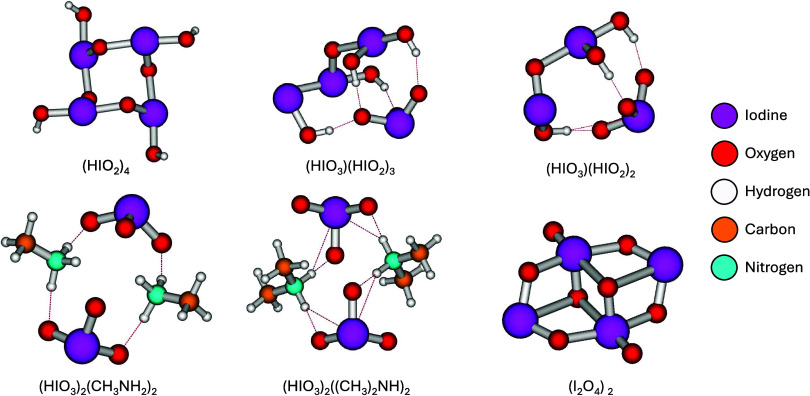
Selected minimum Gibbs free energy structures, calculated at the ZORA-DLPNO–CCSD­(T_0_)/TZVPP//ωB97X-D3BJ/aug-cc-pVTZ-PP level of theory at 298.15 K and 1 atm. Dashed lines indicate hydrogen bonding. Strong X-O bonds have not been drawn on the iodine oxyacid containing structures. These intermolecular I–O bonds are 0.2–0.4Å longer than the intramolecular I–O bonds. The iodine tetroxide dimer taken from our previous study,^39^ and iodine oxyacids are reoptimized structures from Zhang et al.^17^

We have also included the oxide structures from our previous study,^39^ of which the IT dimer (I_2_O_4_)_2_, can be seen in the figure. The general cage-like structure of (I_2_O_4_)_2_ is held together by strong halogen bonds. This crystal-like structure is akin to the one observed for the pure IsA cluster.

### 3.2 Cluster Formation Potential

We report the cluster formation potential of iodine oxyacid clusters, oxyacid-assisted iodine oxide, and iodine oxyacid–amine nucleation assisted by common atmospheric nucleation precursors at 263.15 K in Figure 2a and 283.15 K in Figure 2b. Pure iodine oxyacid–amine nucleation is compared with pure sulfuric acid–amine nucleation in Figure 3. Thermochemical data can be found in Table S1 in the SI, and in the atmospheric cluster database (ACDB).^42^ As discussed in the computational details, our threshold for significant nucleation is 1 cm^–3^ s^–1^; however, due to the limitation of our system size, we would need to observe a formation potential of at least 1 order of magnitude higher to be sure that the nucleation rate is significant.

**Figure 2:**
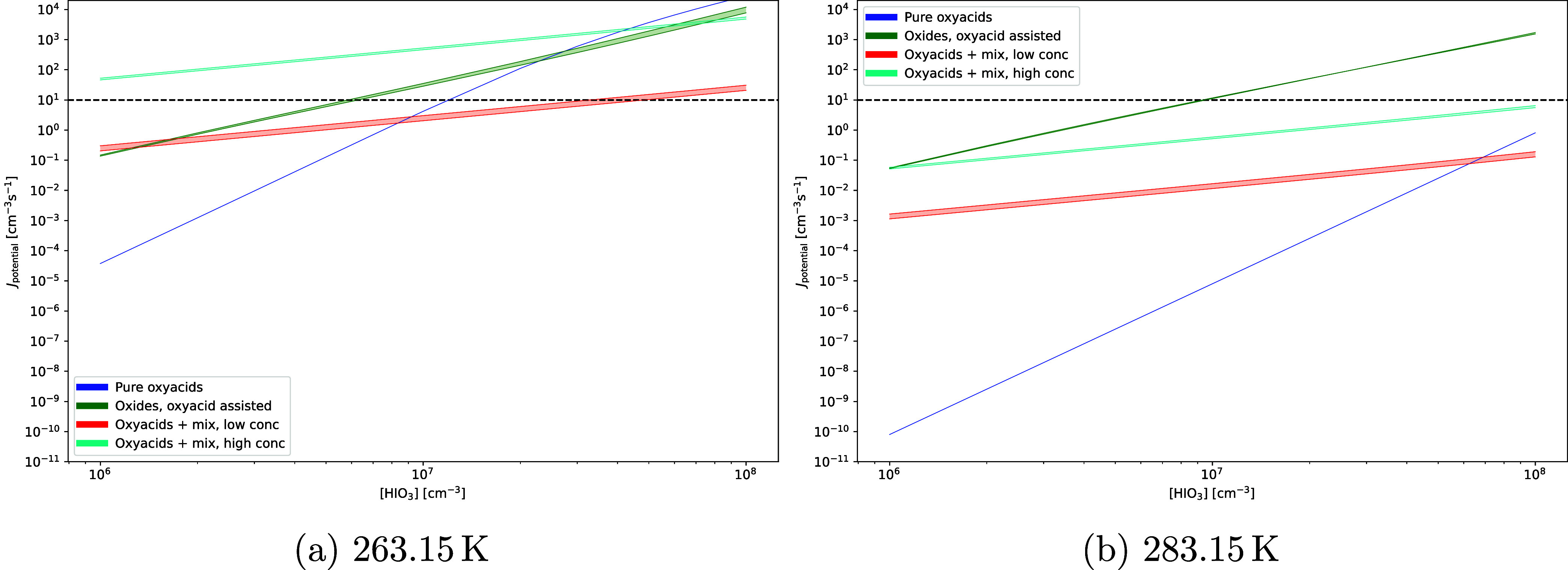
Cluster formation potentials at (a) 263.15 K and (b) 283.15 K for the 3 cluster systems: “Pure oxyacids” with clusters consisting of (IA/IsA)_0–4_, “Oxides, oxyacid assisted” with clusters consisting of (IP/IT)_0–2_(W)_0–3_ and (IP/IT)_1_(IA/IsA)_1_(W)_0–2_, and “Oxyacids + mix” consisting of (IA/IsA)_0–2_(base)_0–2_, where base is defined in the computational details, and consisting of (IA/IsA)_1_(SA/MSA/NA/FA)_1_(base)_0–2_. The shaded area corresponds to relative humidities ranging between 34 and 73%, but note that the iodine oxyacids are run without water. The dashed horizontal line shows the significance threshold of 10 cm^–3^ s^–1^.

**Figure 3:**
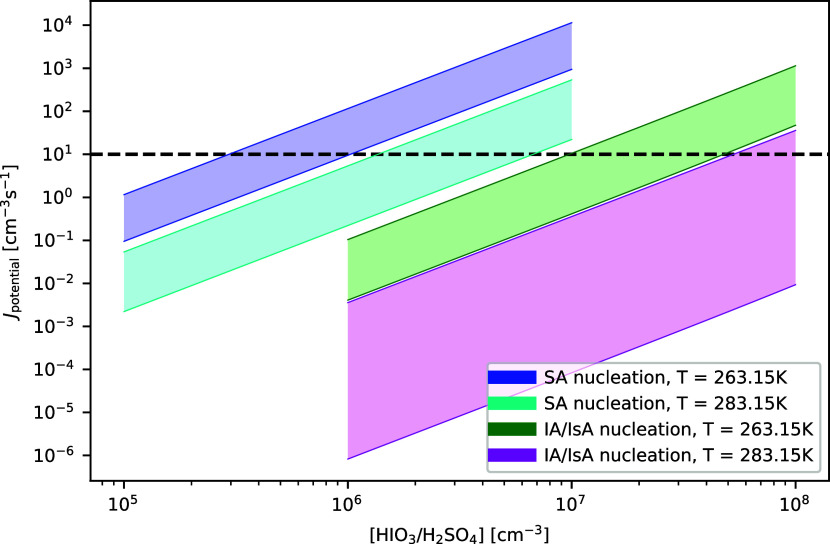
Cluster formation potentials at 263.15 and 283.15 K at a relative humidity of 0% for the 2 cluster systems: SA nucleation given by (SA)_0–2_(base)_0–2_ and IA/IsA nucleation given by (IA/IsA)_0–2_(base)_0–2_, where base is a combination of AM, MA, DMA, and TMA as defined in the computational details. The shaded areas in the figure correspond to the high and low concentrations of amines given in Table 1. The dashed horizontal line shows the significance threshold of 10 cm^–3^ s^–1^.

For the majority of the pure iodine oxyacid clusters, we have reoptimized and recalculated the structures by Zhang et al.^17^ In their study, Zhang et al. treated iodine using a pseudopotential during the calculation of the electronic contribution to the total energy. Thus, they take relativistic contribution to the energy into account implicitly through the construction of the pseudopotential; however, most pseudopotentials are only parametrized for small simple molecules. This means that the accuracy for larger molecules and clusters is uncertain. As shown in our previous benchmark^33^, there is a significant difference of several kcal mol^–1^ in binding energies between explicit and implicit treatment of relativistic contributions. Thus, we find that the nucleation is significantly weakened compared to the numbers reported by Zhang et al.

In Figure 2a, we plotted the cluster formation potential of the three nucleation pathways defined in the computational details. The shaded areas in the figures corresponds to the difference between running the simulation at 34 and 73% relative humidity, except for pure oxyacids, which are run under anhydrous conditions. It can be seen that the cluster formation potential of the pure oxyacids is low at this level of theory, starting at 10^–5^ cm^–3^ s^–1^ for IA concentrations of 10^6^ cm^–3^, only rising above 1 cm^–3^ s^–1^ around 1 × 10^7^ cm^–3^. This is within the upper limit of measured IA concentrations during aerosol bursts,^11^ but, at the high end of normal concentrations^14,15^ On the other hand, the “oxyacid + mixed acids” nucleation pathway, starts around 10^–1^ cm^–3^ s^–1^ at low amine concentrations and starts closer to 10^2^ cm^–3^ s^–1^ at high amine concentrations. However, it exhibits a shallower slope with higher IA concentrations, but in both cases, we see significant nucleation at more common IA concentrations for the “oxyacid + mixed acids” system compared with the “pure oxyacid” nucleation. Finally, we see that the ″oxyacid-assisted oxide” nucleation starts at 10^–1^ cm^–3^ s^–1^, but rises more steeply than the “mixed acid” pathway. Even at low iodine concentrations, it is competitive against the “mixed acid” pathway at low amine concentrations, and it can compete with the “mixed acid” pathway at high amine concentrations given high enough iodine concentrations.

Keeping in mind that the iodine oxide potential nucleation is likely to be underestimated due to the neglect of spin–orbit coupling, we can see that the oxyacid-assisted oxide pathway is competitive against the pure oxyacid pathway, especially at lower concentrations. Noting the overestimation of the nucleation potential due to the small system size, it can be seen that the oxide pathway is a more likely candidate to explain the nucleation rates reported for iodine nucleation by He et al.,^16^ which are reported to be of the order of 10^2^ cm^–3^ s^–1^ at IA concentrations of 10^7^ cm^–3^. Both the oxide and pure oxyacid pathway falls below this number; however, the oxide is both closer, and it is known that the spin–orbit neglect can lead to a significant increase in nucleation. This is despite the relatively low concentrations of the oxides, which are compensated by significantly stronger bonding exhibited by the oxide clusters.

A caveat to this result is that we run the iodic acid simulation results without water, which can, of course, also increase the nucleation rate. Other studies disagree with this conclusion; for example, Zhang et al.^17^ reports good agreement to the CLOUD data reported by He et al.^16^ for a purely iodine oxyacid pathway. However, we believe that this is due to a cancellation of errors, which has several causes. (1) As stated in the cluster formation dynamics section, they study up to a diameter of 1.5 nm, while the measurements are carried out at 1.7 nm. Which means that their calculations overestimate the nucleation rate, with the true rate for that pathway being lower in reality. (2) They do not adequately take into account relativistic effects, which we have previously shown^39^ to lead to an overestimation of several kcal mol^–1^ in the binding energy, thus also several orders of magnitude for the nucleation rate. These limitations in their method can obscure the need for including more nucleation pathways into the mechanism, such as pathways including iodine oxide in the nucleation.

In Figure 2b, the temperature is increased from 263 to 283 K. The cluster formation potentials decrease by many orders of magnitude for the “pure oxyacid” clusters, owing to the relatively weak bonding in the clusters. On the other hand, the “oxide” clusters that exhibit very strong bonding are barely affected by the temperature change. This means that “iodine oxide” nucleation is not limited by evaporation of the initial clusters due to ambient heat, but rather the amount of available oxide in the gas-phase. The “mixed acid” nucleation pathway can be seen to decrease by around 2 orders of magnitude. The high amine concentration scenario is only competitive with “oxide” nucleation at low iodine concentrations.

In Figure 3, we compare the rates of SA–amine nucleation with iodine oxyacid–amine nucleation. The upper and lower bounds of the shaded areas correspond to the applied high and low amine concentrations, which can be seen in Table 1. We let SA, IA, and IsA span over the concentrations usually observed in polar regions: between 10^5^ and 10^7^ cm^–3^ for SA,^15^ and IA ranging from 10^6^ to 10^8^ cm^–3^.^11^ The IsA fraction is set at 3% of IA at 263 K and 1% at 283 K based on the data reported by Zhang et al.^17^ The relative humidity is set to 0%. As noted in the computational details, we only use an enhancement factor for the iodine-driven nucleation and not for the SA-driven nucleation. Thus, our results here will be biased toward favorability of iodine-driven nucleation.

As would be expected given the p*K*_a_ of SA, SA–amine nucleation at 263.15 K is significant, reaching values above 1 cm^–3^ s^–1^ at a SA concentration of 10^5^ cm^–3^ with high amine concentrations, and at a SA concentration of 10^6^ cm^–3^ with low concentrations. Noting the overestimation by 1–2 orders of magnitude due to the limited cluster sizes in the simulation, this means that we should have 10^2^ cm^–3^ s^–1^ or more in the simulation to be certain of significant nucleation. At low temperatures, this threshold is reached at SA concentrations between 1 × 10^6^ to 3 × 10^6^ cm^–3^, depending on the amine concentration. At higher temperatures, this threshold is reached at an SA concentration of 10^7^ cm^–3^ at high amine concentrations. Meanwhile at low amine concentrations, it only reaches a value of 10^1^ cm^–3^ s^–1^ and thus never reaches this threshold.

These concentrations are still well within the range of observed SA concentrations, and the overestimation of the nucleation rates is also mitigated by our neglect of hydration. Thus, our method predicts SA–amine nucleation at 283.15 K as expected, where it is driven by the SA–DMA interaction.

For iodine oxyacid–amine nucleation at 263.15 K, we see that we need high amine and oxyacid concentrations to observe cluster formation potentials of 10^2^ cm^–3^ s^–1^. At 283.15 K, it can be seen that the highest concentrations of amines and oxyacids will result in a cluster formation potential of 10^1^ cm^–3^ s^–1^, but otherwise the cluster formation potential will be low. Comparing the two nucleation pathways, we can see that the changes in amine concentrations has a much larger relative effect on the iodine oxyacid pathway at y283.15 K than any of the others; however, we ascribe this be due to the already low nucleation rates.

From the comparison of the two nucleation pathways, we can see that at the upper limit for the acid concentrations with high amine concentrations, the resulting cluster formation potentials are within 1 order of magnitude of each other at both temperatures. However, this is not the case for the low amine concentrations, where iodine oxyacid–amine nucleation cannot compete with SA–amine nucleation.

Therefore, iodine oxyacid–amine nucleation can be a secondary contributing factor in high amine regimes, even at high SA concentrations. However, without application of the appropriate enhancement factor for SA-driven nucleation, the exact fraction is still to be determined.

In Figure 4, we plotted a scan of the potential nucleation rates of ″oxyacid-assisted oxide” and “pure oxyacid” nucleation. A similar scan of the SA–amine and iodine oxyacid–amine pathways can be found in Figure S4. Figure 4 again shows that significant concentrations of IA, > 10^7^ cm^–3^ are needed to induce significant nucleation if no oxides are present. The exact iodine oxide to iodic acid ratios and how relative humidity/temperature affect it have yet to be studied. Therefore, several points have been marked on the figure, representing iodine oxide:iodic acid ratios of 1, 10, 50, 70, and 100 starting from the left. Inspection of the nucleation path shows that 1:1 and 1:10 ratios yield either very little or no contribution from the iodine oxyacids, except for assisting in growing out of the model via collisions. The dominant outgrowing cluster is the IP–IT dimer cluster. At 1:50, the outgrowing clusters are mixed between the oxides and the oxyacids split roughly half and-half, while at 1:70, it is primarily oxyacids, with a third coming from the oxides. At 1:100, it has flipped completely toward the IA–IsA clusters.

**Figure 4:**
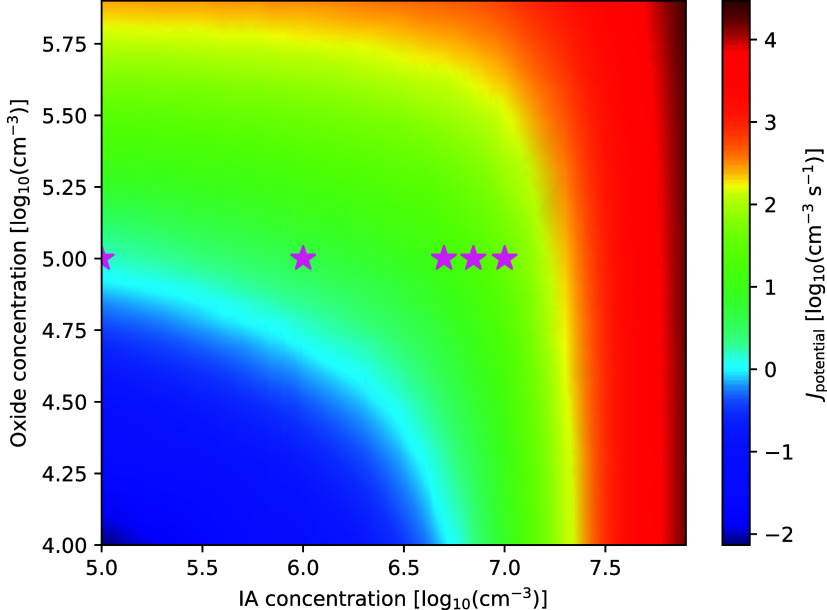
Scan of the potential nucleation rate of the combined oxyacid-assisted oxide and the oxyacid nucleation carried out at 263.15 K. Pink stars denote specific ratios in the scan: 1, 10, 50, 70, 100.

Thus, constraining the ratio between the iodine oxyacids and the iodine oxides in the atmosphere is needed in order to determine how much each pathway contributes to nucleation. It is also important to keep in mind that this scan is run at 263.15 K, where the oxyacids are most favorable relative to the oxides, where the oxyacids fall off significantly at higher temperatures (see Figure 2).

### 3.3 Nucleation Mechanisms

One can look at the major outgrowing clusters for each nucleation path. Tables showing the top three most significant outgrowing clusters for each nucleation path are presented in Tables S2 and S3 in the SI. A selection of these clusters can be seen in Figures 1 and S2. The “pure iodine oxyacid” nucleation path can be seen to be dominated by IsA-heavy clusters under all tested conditions. The outgrowing clusters are (IA)­(IsA)_2_ colliding with other small clusters to grow beyond the border, and they comprise more than 97% of all outgrowing clusters under all tested conditions. As observed by Zhang et al.,^17^ there is an acid–base interaction between IA and IsA, which contributes to the binding energy. Furthermore, we have seen that the IA/IA halogen bond interaction is also a strong contributor to the binding energy. In addition, it can be seen that these types of clusters are stable enough that there is a large enough population of dimers to collide with these trimers and grow out of the simulation box.

In the “iodine oxide” nucleation path, we see that the outgrowing clusters at low temperatures are primarily the “oxyacid-assisted” clusters, mainly IP with either oxyacid (∼12–54%), or the very stable oxide dimers (∼4–31%). This is likely because of the low concentrations of oxides, which lower the likelihood of an oxide–oxide collision, especially at colder temperatures. At higher temperatures, we see an increase in the oxide dimer contributions with a contribution of up to half of the outgrowing clusters; especially, the IT dimer, as these will be very stable against evaporation.

For the “oxyacid + mix,” we see that the relatively high concentrations of FA and NA act as catalysts for nucleation, leading to very high cluster formation potentials. FA and NA was previously shown to bind relatively weakly to iodine species compared to, e.g., SA,^39^ and therefore we believe that the high nucleation rates for the “oxyacid + mix” is due to these additional nucleation precursors pushing them toward stable sizes. This is mainly caused by their large number concentrations, but these clusters are likely to evaporate due to the low binding strength of NA and FA. Therefore, it can be regarded as a simulation artifact.

Finally, for the “iodine oxyacid–amine” nucleation, we see the outgrowing clusters to be completely dominated by DMA (≥98%). This is caused by the relatively low binding free energy between IA and DMA (−7.1 kcal mol^–1^). The IsA and IA bonding (−9.8 kcal mol^–1^) is still stronger; however, the minimum DMA concentrations (1 ppt ∼ 10^7^ cm^–3^) are several orders of magnitude higher than the IsA concentrations (maximum of 3 × 10^6^ cm^–3^). In Tables S2 and S3, it can be seen that increasing the IA concentration (or the corresponding oxide concentrations) from 1 × 10^6^ cm^–3^ to 1 × 10^8^ cm^–3^ does not fundamentally alter the nucleation mechanism. However, one can still observe a shift in the distribution, allowing for an increased contribution from other clusters. This can most strongly be seen for the “oxyacid-assisted oxide” nucleation at 283.15 K, where the major outgrowing clusters at an IA concentration of 10^6^ cm^–3^ are (IP)_1_(IsA)_1_ at 53% and (IT)_2_ at 34% and a minor contribution from (IP)_1_(IT)_1_ at 9%. This growth is primarily achieved through collision with IA, but with noninsignificant (>10%) contribution from collisions with the IT and IP. Increasing the concentration to 10^8^ cm^–3^, the major outgrowing cluster shift to (IP)_1_(IT)_1_ at 30% together with (IP)_1_(IsA)_1_ at 29%, with a minor component of (IT)_2_ at 18%. The top three clusters at low concentrations account for 96% of the nucleation, which decreases to 77% at higher concentrations. For the “oxyacid + mix” pathways, we only observe a slight shift in distributions, and for the “oxyacid” or “oxyacid + amines,” the change is negligible. Likewise, a shift in distributions is observed for 263.15 K, but the same decrease in dominance of the top three outgrowing clusters is not observed, which could be caused by the increased stability at lower temperatures.

### 3.4 Hydration

Figure 2a,b shows part of the effect of hydration due to the limited inclusion of water in the clusters. It can be seen that the effect of varying the relative humidity between 34 and 73% is a modest factor of 2, which is negligible compared to the changes in the cluster formation potential rate due to changes in IA concentration. Therefore, the direct effect of the hydration of these clusters is not significant. However, in this study, we neglect the hydrolysis reaction of the oxide clusters, which have been shown to be reactive with water both experimentally in the bulk-phase^66^ and theoretically in the gas-phase.^41^ The hydrolysis reactions, shown in the reactions in eqs 1 and 2, will lead to a conversion of oxide clusters into oxyacid–water clusters, which has been shown to have a reaction barrier for IP of 7.7 kcal mol^–1^ in the gas-phase.^41^ The rate of the hydrolysis reaction in clusters and the rate of reaction for IT have not currently been determined. Finally, the change in the rate of hydrolysis with an increase in temperature has also not been investigated.

Rörup et al.^36^ have experimentally studied the effect of hydration on iodine-driven nucleation. Under very dry conditions (around 0.008% RH), they observe orders of magnitude larger nucleation rates, which they ascribe to nucleation driven by iodine oxides. When increasing the RH, they observe a large decrease of the nucleation rate, and above a RH of 2%, they find no obvious relation between nucleation rate and RH. We hypothesize that very dry conditions are necessary for nucleation to take place without the rapid conversion of iodine oxides to iodine oxyacids. As can be seen in Figure 2a,b, iodine-oxide-driven nucleation leads to nucleation that is several orders of magnitude greater than purely iodine oxyacid-driven nucleation. However, their study also shows that even dry conditions of 2% RH are enough to “saturate” the hydrolysis, and increasing it further does not lead to significant changes. This indicates that hydrolysis in the clusters is an incredibly rapid and efficient process. However, in Figure 2a,2b, we also show that purely iodine oxyacid-driven nucleation is weak, which would imply that nucleation is kick-started by iodine oxides, which will rapidly hydrolyze in the clusters, but bypasses the initial nucleation barrier that is usually present.

## 4 Conclusions

In this study, we have examined the initial nucleation of iodine species such as HIO_3_, HIO_2_, I_2_O_5_, and I_2_O_4_ together with atmospherically relevant acids and bases such as sulfuric acid, nitric acid, formic acid, ammonia, dimethylamine, trimethylamine, and water. We have explicitly included all relevant iodine oxide–water clusters that are equivalent to the incorporated iodine oxyacid clusters, such that the hydrolysis reactions of the oxides can be incorporated in future studies. By combining clusters from our previous study,^39^ the one by Zhang et al.,^17^ and newly calculated structures, we find both prevalent I–O halogen bonds, acid–base interactions, and hydrogen bonds.

We find that nucleation starting with iodine oxides can provide cluster formation potentials that are more in line with the observed trends in nucleation found by He et al.^16^ in an environmental chamber, compared to nucleation by only iodine oxyacids.^17^ We find that by explicitly taking the scalar relativistic effects into account, a pure iodine oxyacid pathway cannot yield enough nucleation to match the experimentally measured nucleation rates. Thus, more nucleation pathways, involving the iodine oxides, should be included to simulate high enough nucleation. The impact of relative humidity on the nucleation rate is found to be relatively small, where the difference between 34% RH and 73% RH is a factor of 2 in the nucleation rate. Therefore, the primary effect of relative humidity is through the hydrolysis of the iodine oxides; however, additional calculations of the hydrates and the hydrolysis rates are needed to be certain of the exact mechanism.

Finally, we also find that iodine oxyacid–amine nucleation can be competitive with sulfuric acid–ammonia nucleation. However, for the rates to become equivalent, iodic acid concentrations need to be 10 times higher than sulfuric acid concentrations.

## Supplementary Material



## Notes

Structures and thermochemistries of iodine-containing clusters can be found in the ACDB^42^ which is available at: https://github.com/elmjonas/ACDB/tree/master/Articles/engsvang25_iod_ox_oxacids (PDF)
