# Supplementary material for: Iodine Clusters in the Atmosphere II: Cluster Formation Potential of Iodine Oxyacids and Iodine Oxides
Source: ACS Omega. 2025 Jun 3;10(23):24887–96. doi: 10.1021/acsomega.5c02147 (PMC12177774; doi:10.1021/acsomega.5c02147)
Supplement: Supplementary file 1 [file ao5c02147_si_001.pdf]

Supporting Information for: Iodine Clusters in the Atmosphere II: Cluster Formation Potential of Iodine Oxyacids and Iodine Oxides

Morten Engsvang and Jonas Elm*

Department of Chemistry, Aarhus University, Langelandsgade 140, 8000 Aarhus C, Denmark

E-mail: jelm@chem.au.dk

Phone: +45 28938085

S1 Selected Clusters

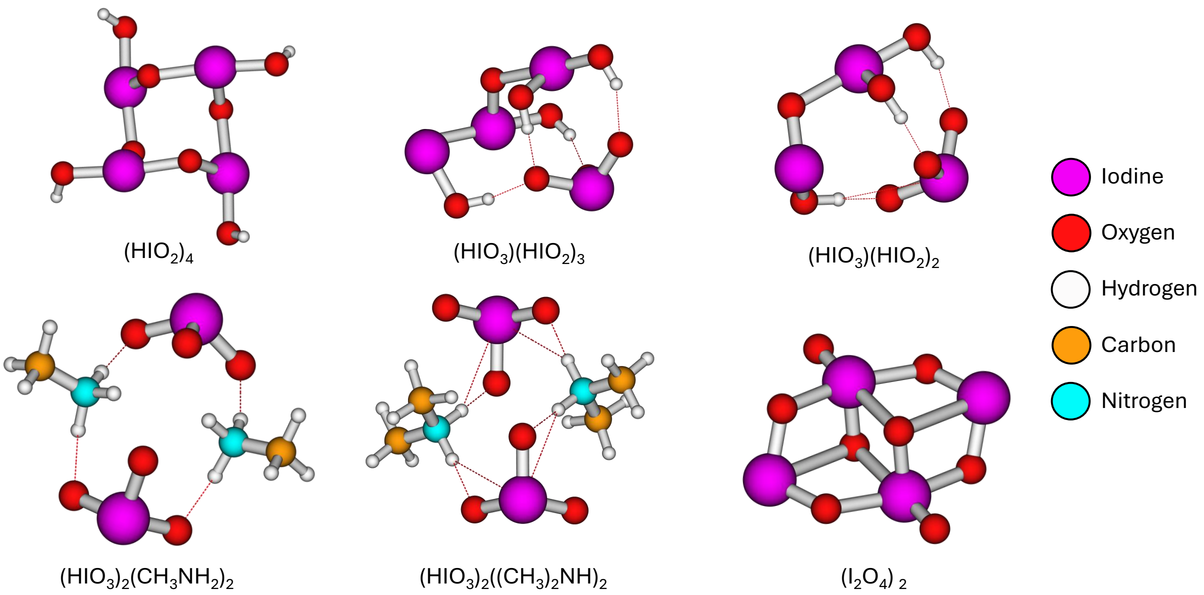

Figure S1: Selected minimum Gibbs free energy structures

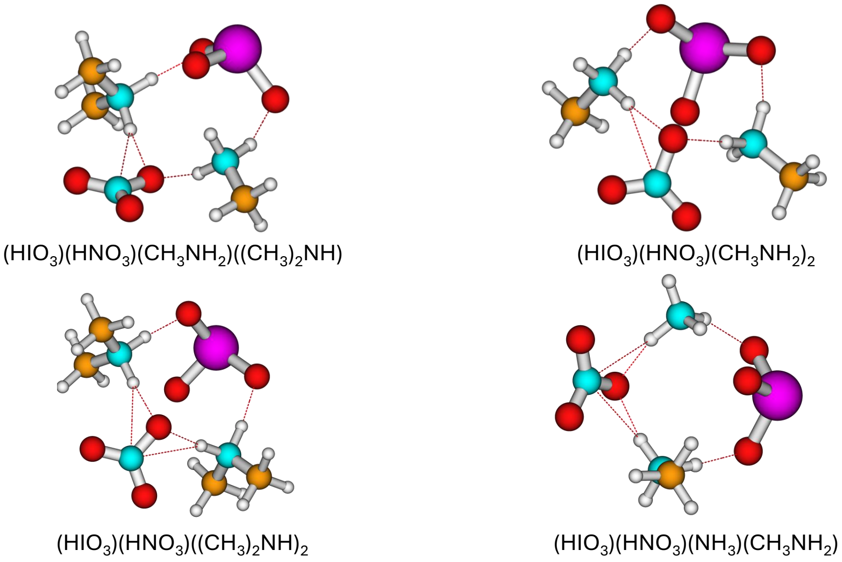

Figure S2: Selected minimum Gibbs free energy structures

S2 Thermochemistry

Table S1: Thermochemistry of the minimum Gibbs free energy structures for each cluster. Calculated at 298.15 K and 1 atm

Cluster	Δ H [kcal mol^{-1}]	ΔS [cal mol^{-1} K^{-1}]	ΔG [kcal mol^{-1}]
3isa	-52.0	-89.4	-25.3
3ica	-39.9	-93.6	-12.0
2ica	-19.9	-47.2	-37.5
2isa	-26.8	-41.3	-14.5
1ip2w	-20.7	-69.2	-0.1
2ip1w	-39.9	-93.0	-12.2
2it1w	-47.0	-91.5	-19.7
1it2w	-33.3	-72.7	-11.6
1isa2w	-22.2	-66.1	-2.5
1ica2w	-18.8	-66.4	1.0
2isa1ma	-34.7	-78.5	-11.3
1ica1isa	-26.9	-45.6	-13.3
1ica2ma	-30.1	-78.3	-6.7
2am1isa	-18.5	-62.7	0.2
1am2isa	-32.3	-76.9	-9.3
1isa2ma	-21.7	-73.7	0.3
1am2ica	-32.5	-80.1	-8.6
2am1ica	-21.8	-64.2	-2.7
2ica1ma	-42.4	-86.3	-16.6
1dma2ica	-46.2	-86.4	-20.4

2dma1isa	-24.9	-75.6	-2.3
1ip1ma1w	-41.2	-78.3	-17.9
1am1ip1w	-34.6	-73.7	-12.6
2isa1tma	-38.8	-82.4	-14.2
1ica2tma	-31.7	-79.2	-8.1
2dma1ica	-34.7	-80.6	-10.6
1am1it1w	-35.6	-72.9	-13.9
2ica1tma	-41.7	-85.3	-16.3
1ip1it1w	-51.7	-90.4	-24.7
1dma2isa	-39.8	-83.0	-15.1
1isa2tma	-26.0	-76.1	-3.3
2ica1isa	-49.3	-94.4	-21.1
1it1ma1w	-39.2	-77.7	-16.1
1ica2isa	-55.3	-91.8	-27.9
1fa1isa1w	-28.4	-74.2	-6.3
1ica1sa1w	-33.5	-77.1	-10.5
1ica1ip1w	-38.8	-85.7	-13.2
1dma1ip1w	-46.7	-78.5	-23.3
1ica1it1w	-42.7	-85.9	-17.1
1isa1ma1w	-23.0	-68.8	-2.5
1ica1ma1w	-29.6	-73.9	-7.6
1am1isa1w	-19.9	-67.1	0.1
1am1ica1w	-24.4	-69.4	-3.7
1ip1isa1w	-45.8	-83.3	-21.0

1dma1it1w	-42.6	-78.2	-19.3
1it1tma1w	-37.6	-77.7	-14.4
1ip1tma1w	-40.6	-77.4	-17.5
1fa1ica1w	-25.8	-74.2	-3.7
1isa1it1w	-44.5	-84.9	-19.2
1isa1sa1w	-36.9	-75.1	-14.5
1isa1ma1sa	-43.1	-83.7	-18.1
1am1ica1sa	-39.5	-79.3	-15.8
1am1ica1ma	-28.5	-73.1	-6.7
1am1isa1ma	-20.7	-69.1	-0.1
1am1isa1sa	-39.7	-74.2	-17.6
1ica1msa1w	-29.5	-80.6	-5.5
1ica1ma1sa	-45.9	-83.7	-20.9
1ica1nta1w	-20.5	-70.3	0.4
1isa1nta1w	-30.3	-72.9	-8.5
1fa1isa1ma	-32.0	-77.5	-8.9
1dma1ica1w	-32.5	-75.3	-10.1
1isa1msa1w	-35.5	-76.5	-12.7
1isa1tma1w	-25.4	-71.7	-4.0
1fa1ica1ma	-34.8	-77.8	-11.6
1ica1tma1w	-27.4	-74.6	-5.2
1dma1isa1w	-25.6	-70.8	-4.5
1am1fa1ica	-28.1	-72.8	-6.4
1am1fa1isa	-30.0	-73.0	-8.2

1am1ica1msa	-38.0	-79.7	-14.2
1am1ica1isa	-39.5	-80.7	-15.4
1am1dma1isa	-23.1	-70.4	-2.1
1isa1sa1tma	-46.0	-81.5	-21.7
1dma1isa1ma	-24.0	-74.6	-1.8
1dma1isa1sa	-46.0	-78.5	-22.6
1ica1ma1msa	-44.7	-84.8	-19.4
1ica1sa1tma	-46.3	-82.6	-21.7
1ica1ma1nta	-33.7	-81.1	-9.5
1ica1ma1tma	-30.4	-78.7	-6.9
1dma1ica1sa	-49.8	-84.1	-24.8
1fa1isa1tma	-34.7	-79.2	-11.1
1fa1ica1tma	-32.8	-79.7	-9.1
1ica1isa1ma	-42.3	-82.9	-17.6
1dma1fa1ica	-38.1	-80.3	-14.1
1dma1fa1isa	-34.6	-79.7	-10.8
1isa1ma1nta	-34.1	-76.1	-11.4
1isa1ma1msa	-42.0	-82.0	-17.5
1am1ica1tma	-28.3	-72.7	-6.6
1am1ica1nta	-26.8	-76.9	-3.9
1am1dma1ica	-31.6	-74.0	-9.5
1am1isa1msa	-37.5	-77.0	-14.5
1am1isa1nta	-32.8	-72.1	-11.3
1am1isa1tma	-24.0	-70.2	-3.0

1dma1ica1ma	-33.5	-79.4	-9.8
1isa1ma1tma	-25.0	-75.0	-2.7
1dma1ica1nta	-37.7	-81.1	-13.5
1ica1msa1tma	-44.2	-83.1	-19.4
1ica1nta1tma	-35.8	-79.7	-12.1
1dma1isa1tma	-26.0	-76.5	-3.1
1dma1isa1msa	-45.6	-82.2	-21.1
1dma1isa1nta	-37.4	-78.3	-14.0
1dma1ica1isa	-45.6	-83.1	-20.8
1ica1isa1tma	-44.0	-86.5	-18.2
1isa1nta1tma	-35.6	-79.3	-12.0
1dma1ica1msa	-45.4	-83.0	-20.6
1dma1ica1tma	-31.3	-80.1	-7.5
1isa1msa1tma	-44.9	-85.7	-19.3
4ica	-65.9	-141.7	-23.6
4isa	-77.2	-136.0	-36.7
2ip2w	-54.7	-125.9	-17.2
1it3w	-43.1	-106.3	-11.4
2it2w	-67.3	-126.2	-29.7
1ip3w	-37.4	-109.7	-4.7
1ica3w	-29.1	-100.4	0.8
1isa3w	-29.4	-101.8	1.0
3ica1w	-52.4	-125.9	-14.9
2am2ica	-48.8	-113.0	-15.1

2ica2ma	-63.1	-122.6	-26.5
2am2isa	-44.6	-106.4	-12.9
2isa2ma	-47.0	-115.2	-12.7
3ica1isa	-72.8	-139.5	-31.2
1am1ip2w	-49.6	-107.8	-17.5
1it2ma1w	-51.8	-114.9	-17.5
1it1ma2w	-50.7	-109.8	-17.9
2am1it1w	-46.9	-105.9	-15.3
2dma2ica	-68.7	-125.7	-31.2
2am1ip1w	-48.0	-107.6	-15.9
2dma2isa	-51.6	-122.5	-15.1
1ip2ma1w	-53.7	-117.2	-18.7
1am1it2w	-46.2	-106.9	-14.3
2ica2tma	-54.0	-126.5	-16.3
1ica3isa	-78.8	-138.7	-37.5
1ip1ma2w	-54.8	-111.0	-21.7
2ica2isa	-77.5	-138.8	-36.1
1ip1it2w	-61.3	-128.5	-23.0
2isa2tma	-49.1	-121.8	-12.8
2isa1ma1w	-46.4	-114.6	-12.2
2dma1ip1w	-67.1	-117.7	-32.0
1fa1ica2w	-36.1	-108.1	-3.9
1am2isa1w	-44.5	-106.3	-12.7
2dma1it1w	-56.3	-119.4	-20.7

1am2ica1w	-49.3	-116.3	-14.6
1dma1it2w	-51.4	-110.1	-18.6
1it1tma2w	-49.0	-111.7	-15.7
1ica1it2w	-51.8	-119.3	-16.2
1fa1isa2w	-52.2	-95.5	-23.8
1ica1ip2w	-47.8	-121.9	-11.5
1ica1ma2w	-42.1	-105.5	-10.7
1ip2tma1w	-52.5	-117.4	-17.5
1it2tma1w	-50.7	-117.8	-15.6
1isa1ma2w	-33.2	-101.9	-2.8
1isa1it2w	-58.0	-120.2	-22.2
1isa1sa2w	-47.7	-106.8	-15.8
1ip1isa2w	-58.1	-115.6	-23.6
1dma1ip2w	-54.6	-112.2	-21.1
2ica1ma1w	-54.8	-119.3	-19.2
1ip1tma2w	-50.6	-111.0	-17.5
1ica1sa2w	-42.2	-111.9	-8.9
1dma2isa1w	-49.5	-115.7	-15.0
1ica1msa2w	-42.0	-117.0	-7.2
1isa1tma2w	-36.1	-105.1	-4.8
1isa1msa2w	-49.5	-113.9	-15.6
1isa2ma1sa	-57.5	-118.3	-22.2
1isa1nta2w	-40.5	-104.9	-9.2
1fa1isa2ma	-40.8	-114.0	-6.8

1fa1ica2ma	-42.0	-115.8	-7.5
2am1isa1sa	-48.5	-103.7	-17.6
1ica1nta2w	-31.4	-105.9	0.1
1ica2ma1sa	-61.1	-125.6	-23.7
2ica1tma1w	-49.9	-121.2	-13.8
2isa1tma1w	-50.2	-116.6	-15.5
1dma2ica1w	-57.4	-120.6	-21.5
1ica1tma2w	-38.1	-108.7	-5.7
1am2ica1ma	-54.5	-118.4	-19.2
2am1fa1ica	-41.4	-108.7	-9.0
1am2isa1ma	-45.7	-111.9	-12.4
2am1fa1isa	-37.5	-103.8	-6.6
2am1ica1sa	-55.5	-115.4	-21.1
1am1ip1ma1w	-51.7	-111.5	-18.4
1am1it1ma1w	-50.4	-111.1	-17.3
2dma1ica1sa	-75.5	-125.2	-38.1
2am1isa1msa	-52.4	-113.0	-18.7
2am1isa1nta	-41.5	-103.2	-10.7
1isa1ma2tma	-32.4	-112.3	1.1
1dma2ica1ma	-60.2	-127.6	-22.2
1ica2ma1nta	-55.9	-120.0	-20.1
1ica1sa2tma	-62.4	-123.2	-25.6
2ica1ma1tma	-56.0	-127.8	-17.9
2dma1isa1ma	-34.7	-113.4	-0.9

2am1ica1isa	-50.0	-111.6	-16.8
2am1ica1msa	-52.7	-115.1	-18.4
2am1ica1nta	-43.6	-111.1	-10.5
1isa2ma1msa	-59.2	-124.3	-22.1
1isa2ma1nta	-45.0	-113.9	-11.1
1ica2ma1msa	-64.0	-121.8	-27.7
1am1dma2ica	-51.5	-119.4	-15.9
1am1dma2isa	-48.4	-114.6	-14.2
1isa1sa2tma	-60.3	-121.8	-24.0
2dma1isa1sa	-65.4	-121.4	-29.2
2dma1fa1ica	-45.3	-119.0	-9.8
2dma1fa1isa	-47.3	-118.3	-12.1
2dma1ica1ma	-38.1	-116.6	-3.3
1am2ica1tma	-54.1	-122.7	-17.5
1am2isa1tma	-48.7	-114.4	-14.5
2isa1ma1tma	-48.6	-120.2	-12.8
1fa1isa2tma	-44.1	-116.7	-9.3
1fa1ica2tma	-46.9	-119.8	-11.1
1dma2isa1ma	-49.8	-121.6	-13.5
1ica1isa2ma	-55.0	-121.0	-18.9
1fa1isa1ma1w	-41.2	-109.0	-8.7
1ica1isa2tma	-47.5	-126.1	-9.9
2dma1ica1msa	-70.3	-125.1	-33.0
1am1dma1it1w	-53.7	-113.4	-19.9

2dma1ica1isa	-59.1	-124.0	-22.1
2dma1isa1nta	-55.2	-121.8	-18.8
2dma1isa1msa	-63.2	-124.6	-26.1
1am1fa1isa1w	-38.8	-106.1	-7.2
1ip1ma1tma1w	-53.2	-118.8	-17.8
1ica1msa2tma	-58.0	-124.9	-20.7
1ica1ma1sa1w	-56.7	-115.7	-22.2
1ica1nta2tma	-51.1	-122.8	-14.5
1am1fa1ica1w	-42.0	-109.7	-9.3
1am1ica1sa1w	-51.1	-112.3	-17.6
1dma1ip1ma1w	-64.4	-116.1	-29.8
1it1ma1tma1w	-51.3	-116.3	-16.7
2dma1ica1nta	-63.8	-121.5	-27.6
1isa1msa2tma	-59.2	-125.0	-21.9
1isa1ma1sa1w	-54.5	-116.0	-19.9
1isa1nta2tma	-48.2	-117.8	-13.1
1am1isa1sa1w	-49.0	-105.9	-17.5
1am1dma1ip1w	-55.3	-111.6	-22.1
1am1ip1tma1w	-48.5	-115.5	-14.1
1am1it1tma1w	-49.7	-112.5	-16.1
1dma1it1ma1w	-55.1	-117.1	-20.2
1dma2ica1tma	-59.7	-127.8	-21.6
1dma2isa1tma	-51.5	-120.9	-15.5
1fa1ica1ma1w	-41.4	-112.1	-8.0

1am1ica1nta1w	-41.7	-111.1	-8.6
1am1isa1ma1sa	-53.3	-108.3	-21.1
1fa1isa1tma1w	-51.9	-99.2	-22.3
1fa1ica1tma1w	-43.8	-111.9	-10.5
1dma1isa1ma1w	-35.9	-108.6	-3.6
1dma1isa1sa1w	-57.9	-116.5	-23.1
1am1fa1isa1ma	-41.9	-110.8	-8.9
1ica1sa1tma1w	-56.0	-116.3	-21.3
1dma1ip1tma1w	-58.6	-120.3	-22.7
1am1ica1isa1w	-49.8	-113.0	-16.1
1am1ica1ma1sa	-57.3	-114.8	-23.1
1am1fa1ica1ma	-40.5	-109.5	-7.9
1isa1sa1tma1w	-55.6	-113.8	-21.7
1am1isa1msa1w	-51.2	-112.9	-17.6
1am1ica1msa1w	-50.6	-113.6	-16.8
1ica1isa1ma1w	-53.5	-117.1	-18.6
1ica1ma1nta1w	-47.2	-114.1	-13.2
1dma1it1tma1w	-54.7	-118.5	-19.4
1ica1ma1msa1w	-54.6	-116.4	-19.9
1ica1ma1tma1w	-39.9	-112.8	-6.3
1dma1ica1ma1w	-38.5	-112.6	-4.9
1dma1fa1ica1w	-46.8	-114.4	-12.7
1isa1ma1nta1w	-44.2	-111.3	-11.0
1isa1ma1msa1w	-55.2	-116.4	-20.5

1isa1ma1tma1w	-36.6	-105.9	-5.0
1am1isa1nta1w	-41.6	-103.7	-10.7
1dma1fa1isa1w	-52.5	-101.1	-22.4
1dma1ica1sa1w	-59.1	-117.3	-24.2
1am1isa1sa1tma	-55.5	-114.4	-21.4
1am1ica1ma1msa	-58.4	-118.6	-23.1
1am1dma1fa1isa	-45.1	-112.5	-11.6
1isa1nta1tma1w	-44.7	-112.7	-11.1
1dma1ica1nta1w	-49.4	-115.4	-15.0
1am1fa1ica1tma	-43.3	-113.0	-9.6
1am1fa1isa1tma	-41.2	-109.6	-8.5
1am1dma1isa1sa	-56.7	-116.1	-22.1
1am1dma1fa1ica	-42.8	-112.6	-9.3
1isa1msa1tma1w	-56.1	-118.1	-20.9
1fa1ica1ma1tma	-45.4	-119.1	-9.9
1dma1isa1msa1w	-58.9	-120.0	-23.1
1ica1ma1sa1tma	-68.0	-124.1	-31.0
1am1ica1ma1nta	-50.0	-115.6	-15.6
1dma1ica1ma1sa	-70.6	-124.3	-33.6
1dma1ica1msa1w	-56.8	-119.0	-21.3
1dma1ica1isa1w	-57.1	-120.2	-21.2
1dma1fa1ica1ma	-44.9	-119.8	-9.2
1dma1fa1isa1ma	-46.6	-118.6	-11.2
1dma1isa1nta1w	-48.1	-111.0	-15.0

1dma1isa1ma1sa	-60.3	-119.7	-24.6
1ica1msa1tma1w	-51.8	-114.4	-17.7
1ica1nta1tma1w	-45.5	-111.8	-12.2
1am1ica1sa1tma	-61.6	-119.7	-26.0
1am1isa1ma1nta	-43.5	-107.7	-11.4
1isa1ma1sa1tma	-60.2	-118.9	-24.7
1am1isa1ma1msa	-56.0	-116.4	-21.3
1am1dma1ica1sa	-61.1	-117.3	-26.2
1ica1isa1tma1w	-53.1	-119.3	-17.6
1fa1isa1ma1tma	-42.3	-113.7	-8.4
1am1ica1isa1ma	-53.3	-116.4	-18.6
1am1dma1ica1nta	-54.0	-115.8	-19.5
1am1dma1ica1msa	-61.5	-120.1	-25.7
1ica1isa1ma1tma	-53.4	-124.6	-16.3
1isa1ma1msa1tma	-57.0	-123.0	-20.3
1am1isa1msa1tma	-53.6	-117.1	-18.7
1am1isa1nta1tma	-46.2	-111.9	-12.8
1isa1ma1nta1tma	-49.6	-119.6	-13.9
1ica1ma1nta1tma	-49.3	-121.7	-13.0
1am1ica1nta1tma	-48.1	-114.5	-14.0
1dma1fa1isa1tma	-44.6	-117.6	-9.5
1am1ica1msa1tma	-53.7	-120.5	-17.8
1dma1ica1ma1msa	-67.3	-123.7	-30.4
1dma1ica1ma1nta	-59.9	-120.5	-24.0

1dma1isa1ma1nta	-51.6	-120.1	-15.8
1dma1isa1ma1msa	-61.5	-124.0	-24.6
1dma1isa1sa1tma	-63.7	-120.4	-27.8
1dma1ica1sa1tma	-66.2	-124.7	-29.0
1dma1fa1ica1tma	-44.7	-118.6	-9.4
1am1ica1isa1tma	-52.7	-117.0	-17.8
1am1dma1ica1isa	-56.9	-118.6	-21.5
1am1dma1isa1nta	-49.1	-114.7	-14.9
1am1dma1isa1msa	-59.4	-118.6	-24.0
1dma1ica1isa1ma	-58.3	-123.4	-21.6
1ica1ma1msa1tma	-58.7	-125.7	-21.2
1dma1isa1msa1tma	-61.6	-123.6	-24.8
1dma1ica1isa1tma	-56.7	-126.5	-19.0
1dma1ica1nta1tma	-57.4	-120.5	-21.5
1dma1isa1nta1tma	-53.2	-120.4	-17.3
1dma1ica1msa1tma	-61.6	-126.1	-24.0
2it3w	-76.9	-162.2	-28.6
2ip3w	-66.7	-159.6	-19.1
1it4w	-53.3	-141.8	-11.1
1ip4w	-51.6	-143.2	-8.9
1isa4w	-39.9	-132.4	-0.5
3ica2w	-64.2	-163.8	-15.3
1ica4w	-39.3	-134.5	0.8
4ica1w	-75.7	-181.3	-21.7

1ip1it3w	-72.4	-162.0	-24.1
1ip1isa3w	-70.1	-153.6	-24.3
1ica1it3w	-64.4	-155.5	-18.1
1ica1ip3w	-58.7	-154.7	-12.6
1isa1it3w	-70.0	-154.8	-23.8
2ip4w	-77.5	-197.3	-18.6
2it4w	-85.9	-201.1	-26.0
4ica2w	-86.4	-219.1	-21.1
3ica3w	-68.7	-193.8	-10.9
1ip1it4w	-83.3	-197.8	-24.4
1isa1it4w	-79.2	-188.7	-22.9
1ica1it4w	-72.1	-190.3	-15.4
1ica1ip4w	-70.4	-189.3	-14.0
1ip1isa4w	-79.6	-186.4	-24.0
4ica3w	-93.6	-244.4	-20.7
3ica4w	-81.3	-229.0	-13.0
4ica4w	-104.2	-282.1	-20.1
1ica3isa4w	-120.2	-278.2	-37.3

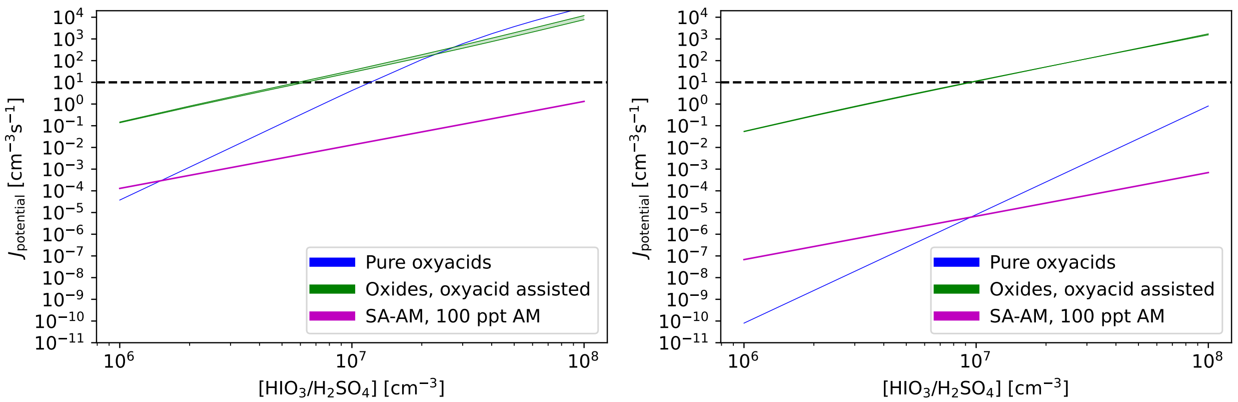

(a) 263.15 K (b) 283.15 K

Figure S3: Cluster formation potentials at different temperatures for the 3 cluster systems: "Pure oxyacids" with clusters consisting of: $(IA/IsA)_{0\text{-}4}$, "Oxides, oxyacid assisted" with clusters consisting of: $(IP/IT)_{0\text{-}2}(W)_{0\text{-}3}$ and $(IP/IT)_1(IA/IsA)_1(W)_{0\text{-}2}$, and "SA-AM, 100 ppt" consisting of: $(SA)_{0\text{-}2}(AM)_{0\text{-}2}$, with 100 ppt AM. The shaded area corresponds to relative humidities ranging between 34% and 73%, however, the cluster formation potential for SA-AM is calculated without water.

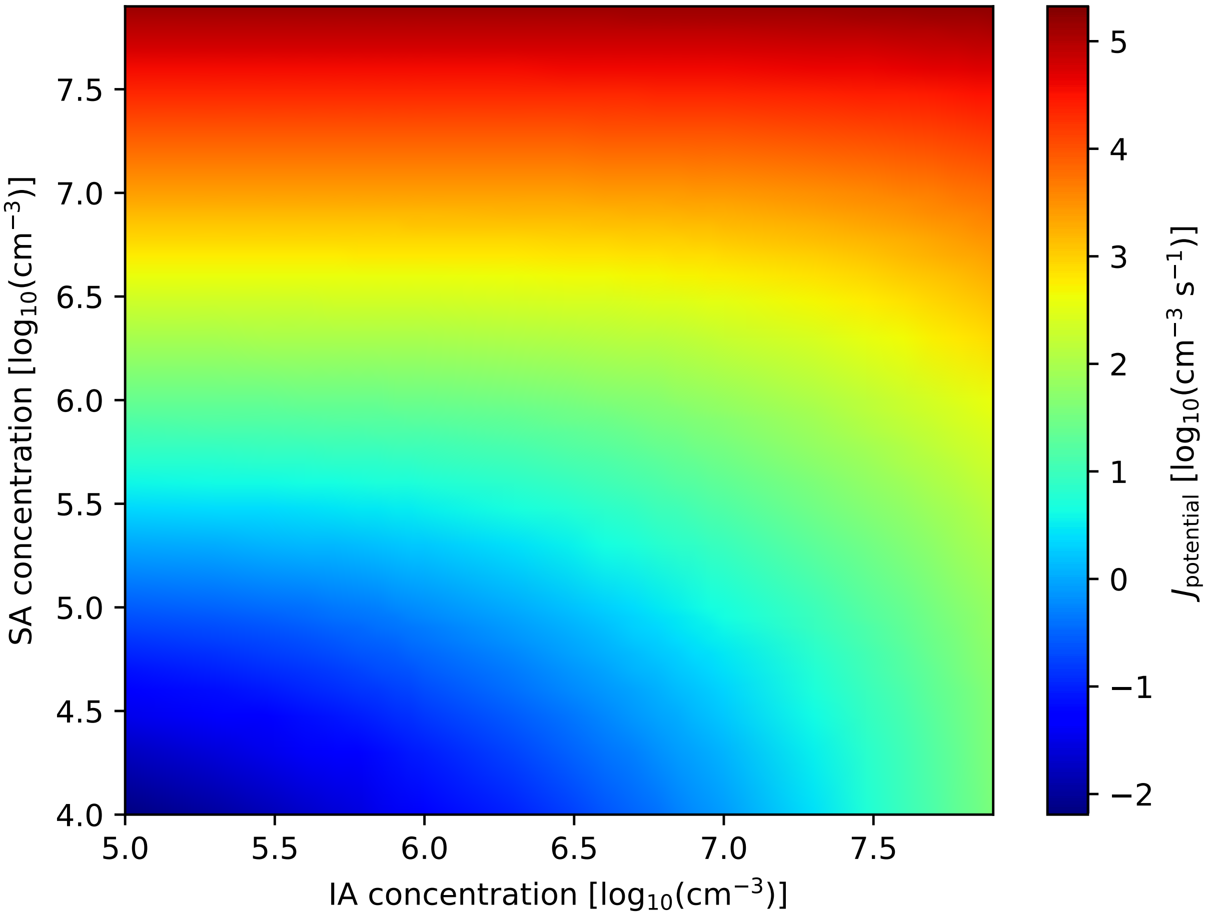

Figure S4: Scan of the potential nucleation rate of the combined SA–amine and iodine oxyacid–amine nucleation carried out at 263.15 K.

S3 Outgrowing Clusters

Table S2: Calculated at 73% RH (pure oxyacid is anhydrous) and 263.15 K.

Pure Oxyacids		
10^6 cm^{-3}	10^7 cm^{-3}	10^8 cm^{-3}
1ica2isa (99%)	1ica2isa (99%)	1ica2isa (99%)
-	-	-
-	-	-
Oxides, oxyacid assisted		
10^6 cm^{-3}	10^7 cm^{-3}	10^8 cm^{-3}
1ip1isa (54%)	1ip1isa (38%)	1ica1ip (73%)
1ip1it (19%)	1ica1ip (30%)	1ip1isa (12%)
2it (12%)	1ip1it (14%)	1ip1it (4%)
Oxyacids + mix, low conc.		
10^6 cm^{-3}	10^7 cm^{-3}	10^8 cm^{-3}
1dma1ica1ma1nta (57%)	1dma1ica1ma1nta (57%)	1dma1ica1ma1nta (56%)
1ica2ma1nta (33%)	1ica2ma1nta (33%)	1ica2ma1nta (33%)
2dma1ica1nta (5%)	2dma1ica1nta (5%)	2dma1ica1nta (5%)
Oxyacids + mix, high conc.		
10^6 cm^{-3}	10^7 cm^{-3}	10^8 cm^{-3}
1am1dma1ica1nta (86%)	1am1dma1ica1nta (85%)	1am1dma1ica1nta (80%)
1am1ica1ma1nta (2%)	1am1ica1ma1nta (2%)	2am2ica(3%)
-	-	1am1ica1ma1nta (2%)
Oxyacids with amines, low conc.		
10^6 cm^{-3}	10^7 cm^{-3}	10^8 cm^{-3}
2dma2ica (100%)	2dma2ica (99%)	2dma2ica (100%)
-	-	-
-	-	-
Oxyacids with amines, high conc.		
10^6 cm^{-3}	10^7 cm^{-3}	10^8 cm^{-3}
2dma2ica (98%)	2dma2ica (98%)	2dma2ica (98%)
2ica2ma (2%)	-	-
-	-	-

Table S3: Calculated at 73% RH (pure oxyacid is anhydrous) and 283.15 K.

Pure Oxyacids		
10^6 cm^{-3}	10^7 cm^{-3}	10^8 cm^{-3}
1ica2isa (98%)	1ica2isa (98%)	1ica2isa (97%)
-	-	-
-	-	-
Oxides, oxyacid assisted		
10^6 cm^{-3}	10^7 cm^{-3}	10^8 cm^{-3}
1ip1isa (53%)	1ip1isa (43%)	1ip1it (30%)
2it (34%)	2it (26%)	1ip1isa (29%)
1ip1it (9%)	1ip1it (25%)	2it (18%)
Oxyacids + mix, low conc.		
10^6 cm^{-3}	10^7 cm^{-3}	10^8 cm^{-3}
1dma1ica1ma1nta (55%)	1dma1ica1ma1nta (54%)	1dma1ica1ma1nta (47%)
1ica2ma1nta (33%)	1ica2ma1nta (33%)	1ica2ma1nta (28%)
2dma1ica1nta (6%)	2dma1ica1nta (6%)	2ica2ma (12%)
Oxyacids + mix, high conc.		
10^6 cm^{-3}	10^7 cm^{-3}	10^8 cm^{-3}
1am1dma1ica1nta (53%)	1am1dma1ica1nta (53%)	1am1dma1ica1nta (48%)
1dma1ica1ma1nta (7%)	1dma1ica1ma1nta (7%)	1dma1ica1ma1nta (6%)
2dma1ica1nta (7%)	2dma1ica1nta (7%)	2dma1ica1nta (6%)
Oxyacids with amines, low conc.		
10^6 cm^{-3}	10^7 cm^{-3}	10^8 cm^{-3}
2dma2ica (100%)	2dma2ica (99%)	2dma2ica (100%)
-	-	-
-	-	-
Oxyacids with amines, high conc.		
10^6 cm^{-3}	10^7 cm^{-3}	10^8 cm^{-3}
2dma2ica (100%)	2dma2ica (100%)	2dma2ica (100%)
-	-	-
-	-	-
